# Comparative Analysis of T-Cell Signatures and Astroglial Reactivity in Parkinson’s Pathology Across Animal Models with Distinct Regenerative Capacities

**DOI:** 10.3390/ijms27020965

**Published:** 2026-01-18

**Authors:** Simona Intonti, Volker Enzmann, Amalia Perna, Ferdinando Spagnolo, Claudia Curcio, Federica Maria Conedera

**Affiliations:** 1 Department of Molecular Biotechnology and Health Sciences, University of Turin, 10126 Turin, Italy; simona.intonti@unito.it (S.I.); ferdinando.spagno57@edu.unito.it (F.S.); 2 Molecular Biotechnology Center, University of Turin, 10126 Turin, Italy; 3 Department of Ophthalmology, Bern University Hospital, 3010 Bern, Switzerland; volker.enzmann@insel.ch; 4 Department for BioMedical Research, University of Bern, 3008 Bern, Switzerland; 5 Department of Pathology, Stanford University, 300 Pasteur Dr., Stanford, CA 94305, USA; amaliap@stanford.edu; 6 School of Advanced Defence Studies, Defence Research & Analysis Institute, Piazza della Rovere 83, 00165 Rome, Italy; 7 Defence Institute for Biomedical Sciences, Via Santo Stefano Rotondo 4, 00184 Rome, Italy; 8 Department for BioMedical Research, Immunology RIA, Inselspital, University of Bern, 3010 Bern, Switzerland; federica.conedera@unibe.ch

**Keywords:** Parkinson’s disease, neurodegeneration, T-cells, astrogliosis, cross-species comparison

## Abstract

Parkinson’s disease (PD) is a progressive neurodegenerative disorder characterized by the selective loss of dopaminergic (DAergic) neurons in the substantia nigra (SN) and the accumulation of misfolded α-synuclein (aSyn). In addition to neuronal pathology, activated microglia are recognized as key mediators of the neuroinflammatory milieu in PD, contributing to DAergic neuron vulnerability. Emerging evidence suggests that the immune system, particularly T-cell-mediated responses, plays a key role in the pathogenesis of PD. However, the heterogeneity of these immune responses across species and preclinical models with varying regenerative capacities remains poorly understood. A comparative analysis of T-cell infiltration, astroglial reactivity, and DAergic neuronal loss across multiple models and species was performed. These included acute DAergic degeneration induced by 1-methyl-4-phenyl-1,2,3,6-tetrahydropyridine (MPTP), genetically modified mice with accumulation of aSyn (Thy1-aSyn L61 model), adult zebrafish exposed to MPTP-induced neurotoxicity and human post-mortem midbrain tissue obtained from PD patients. Zebrafish exhibited transient DAergic neurodegeneration, followed by neuronal regeneration and temporary CD4^+^ T-cell infiltration accompanied by an astroglial response and activation of microglia. In contrast, MPTP-treated mice showed a permanent neuronal loss, marked microglial activation, increased astrogliosis and CD8^+^ T-cell infiltration that was negatively correlated with neuronal survival. By contrast, L61 mice exhibited progressive aSyn accumulation with chronic astrogliosis, mild activation of microglia and CD4^+^ T-cell infiltration not directly linked to neuronal loss. Unlike age-matched controls, the SN from PD brains exhibited DAergic degeneration, aSyn aggregation, and elevated CD3^+^ T-cell infiltration, and increased microglial activation. These changes correlated with neuronal loss and aSyn burden. These findings emphasize the species- and model-specific immune profiles underlying PD pathology. Our results reveal that CD4^+^ T-cells contribute to neuronal regeneration following injury in zebrafish. This process is absent in the MPTP and L61 mouse models, which are instead driven by CD8^+^ or CD4^+^, respectively. This work underscores the potential of targeted immunomodulation aimed at T cell–glial interactions to slow neurodegeneration and promote repair in PD.

## 1. Introduction

Neurodegenerative diseases are a group of disorders that affect the brain and nervous system, typically characterized by the progressive loss and dysfunction of neurons [1]. Among progressive neurodegenerative diseases, Parkinson’s disease (PD) is the second most common condition [2]. As the human population ages, the prevalence of PD is expected to rise significantly, with an estimated 25.2 million PD patients worldwide by 2050 [3].

PD is characterized by the loss of dopaminergic (DAergic) neurons in the substantia nigra (SN) and the accumulation of misfolded α-synuclein (aSyn), leading to motor and non-motor deficits in affected individuals [4]. While the pathogenesis of PD is multifactorial, mounting evidence highlights the critical involvement of the immune system, particularly T-cell-mediated responses, in modulating neuronal degeneration and disease progression [5,6]. In particular, microglial activation is recognized as a pivotal driver of neuroinflammation in PD, promoting dopaminergic neuron vulnerability via the release of pro-inflammatory cytokines, reactive oxygen and nitrogen species, and other neurotoxic mediators [7]. Concurrently, T-cell-mediated immune responses are increasingly appreciated as important regulators of microglial activation and contributors to nigrostriatal degeneration [7]. However, the heterogeneity of T-cell responses across different animal models and species with varying regenerative capacities remains poorly understood [8]. Recently, not only microglia but also astrocytes have emerged as key contributors to PD pathology. In zebrafish, astrocyte-like radial glial cells respond to injury by actively supporting neuronal regeneration, providing a strong rationale for examining astroglial responses in comparative studies of PD [9]. Glial cells, however, have a dual role in the repair process. In regenerative species, such as zebrafish, they enhance neuronal survival and regrowth, whereas in mammals, including mice, they frequently drive glial scar formation, thereby restricting axonal regeneration [10].

To induce PD-like features, we inject 1-methyl-4-phenyl-1,2,3,6-tetrahydropyridine (MPTP) in zebrafish and mice. This toxin-based model has long been a cornerstone of PD research due to its high reproducibility and ability to replicate some of the key neuropathological characteristics of the human condition, including the selective loss of DAergic neurons and neuroinflammation [11,12]. In zebrafish, MPTP-induced neuron loss was followed by activation of the brain and retinal regenerative programs, which is characteristic of this species. In contrast, the same toxin caused an acute and irreversible degeneration of DAergic neurons in mice, reflecting the limited regenerative potential of the mammalian brain [12,13,14].

While the MPTP model reproduces the DAergic neurodegeneration observed in PD, it does not capture the progressive aSyn proteinopathy that is a defining feature of the human disorder. To address this limitation, we also used 4-month-old Thy1-aSyn (L61) transgenic mice that overexpress human aSyn but do not yet exhibit DAergic degeneration. Although aSyn overexpression results in the early accumulation of proteins and alterations to synapses, motor dysfunction and loss of DAergic neurons usually become apparent after the age of 14 months [15]. Indeed, as the mice age, they develop widespread aSyn aggregates, particularly in cortical and subcortical regions, along with neuroinflammatory responses and impaired dopamine neurotransmission [16]. Unlike the acute and rapid neuronal loss observed in MPTP-treated animals, the L61 model recapitulates the slow, cumulative, and aSyn-driven pathology of human PD [17]. Together, these models offer a complementary framework for investigating how immune responses, astroglial activation and regenerative capacity vary according to the nature and timing of the underlying injury. Lastly, to relate these experimental findings to human disease, we also examined post-mortem PD brains, where aSyn pathology is accompanied by immune activation. Building on emerging evidence for adaptive immune mechanisms in PD [18], we hypothesize that T cell infiltration of the nigrostriatal system drives the neuroinflammatory milieu, contributing to disease progression. T cells may exacerbate neurodegeneration, and modulating their activity is a promising way to slow PD progression. To investigate this, we performed a comparative analysis of T-cell infiltration, phenotype, and spatial distribution in established mammalian and non-mammalian PD models, as well as in post-mortem PD midbrain tissue, with the specific goal of characterizing tissue-resident immune–glial interactions within the neurodegenerative microenvironment rather than providing a comprehensive assessment of systemic immunity. Our findings suggest that model- and species-specific patterns of adaptive T-cell subset composition (CD4 versus CD8) and GFAP-positive astroglial reactivity are associated with regenerative capacity and the nature of the pathogenic driver (acute MPTP intoxication versus chronic α-synucleinopathy). Furthermore, these immune–glial states appear to correspond with different dopaminergic outcomes (degeneration versus recovery). We systematically profiled T cells in the substantia nigra of MPTP-treated mice and zebrafish to capture toxin-induced injury, as well as in L61 transgenic mice to model chronic α-synucleinopathy. We then integrated these experimental findings with observations from human PD specimens to delineate model- and species-specific patterns of T-cell involvement, and to assess the contribution of immune repertoire and regenerative capacity to the heterogeneity of T-cell responses in PD. Our cross-species approach provides new insights into T cell-mediated disease mechanisms and may inform the development of targeted immunomodulatory strategies relevant to PD.

## 2. Results

### 2.1. Bibliographic Data Mining

**Figure 1:**
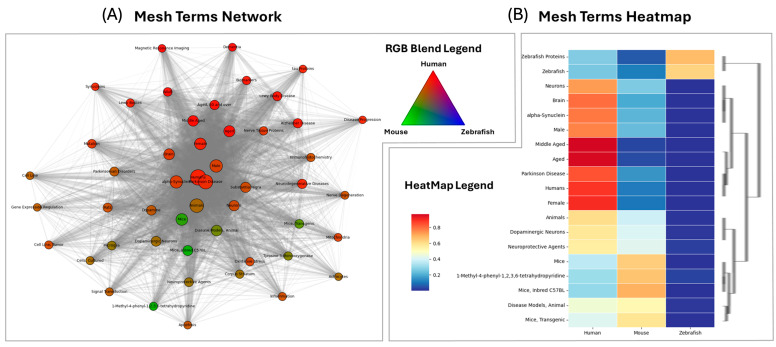
MeSH terms bibliographic relationships in Parkinson’s Disease models. Comparative analysis of the occurrences of MeSH terms associated with Parkinson’s Disease in the main experimental models (Human, Mouse, Zebrafish). (**A**) The co-occurrence graph highlighting the relationships between the most representative terms (nodes RGB Blended according to their relative prevalence in the three species: red = Human, green = Mouse, blue = Zebrafish); (**B**) Clustered heatmap with dendrogram showing the normalized distribution of the selected terms. Species-specific enrichment patterns can conceptually cluster clinical and neuropathological terms (Human), neuroinflammatory markers and transgenic models (Mouse), and regeneration and developmental terms (Zebrafish).

This section provides a scoping, metadata-driven map to contextualize the experimental work. Systematic parsing of 35,502 Parkinson-related PubMed records yielded structured metadata for abstracts, authors and MeSH/keyword fields, exposing a species distribution dominated by human (26,954), mouse (2558), comparative (5827) and zebrafish (163) studies and highlighting “Parkinson’s disease”, “α–synuclein”, “neuroinflammation” and “microglia” as the most frequent descriptors (Figure 1). This pipeline first leverages the structured MEDLINE layer (MeSH/keywords) to derive species level descriptor enrichment and then complements it with LLM-assisted mining of unstructured titles/abstracts to capture relationships not encoded in indexing. Unsupervised text–mining (TF–IDF + PCA + K–means) resolved five stable thematic clusters and, through expanded term matching, quantified immune-degeneration markers across the corpus (GFAP = 1596, CD4 = 458, CD8 = 891, DA–loss = 6951 hits), while silhouette analysis (k = 5, score ≈ 0.998) confirmed cluster separability. Qualitative clustering delineated species-specific “information clouds” that can guide targeted literature searches and thereby refine subsequent content analysis (Figure 1). A neuro-immunology-specialised Deepseek (executed locally via Ollama) LLM prompt then assisted the extraction and manual curation of a high-confidence subset (n ≈ 380 structured records), showing a mouse-centred evidence base (58.3%) and revealing that only 23.1% explicitly report multivariate GFAP–CD4/CD8–CD8-dopaminergic-loss patterns, just 14 papers examine GFAP’s reliability as an astroglial marker, and 154 link CD4/CD8 infiltration to DA–neuron loss; full methodological details, cluster composition and PMID lists are provided in the “Bibliographic Data Mining” section of the Supplementary Materials. All model outputs were deterministically parsed and human curated to mitigate interpretive ambiguity; the LLM functioned strictly as decision support rather than as an autonomous reviewer.

### 2.2. Acute Neurodegeneration in the Zebrafish Model of PD

**Figure 2:**
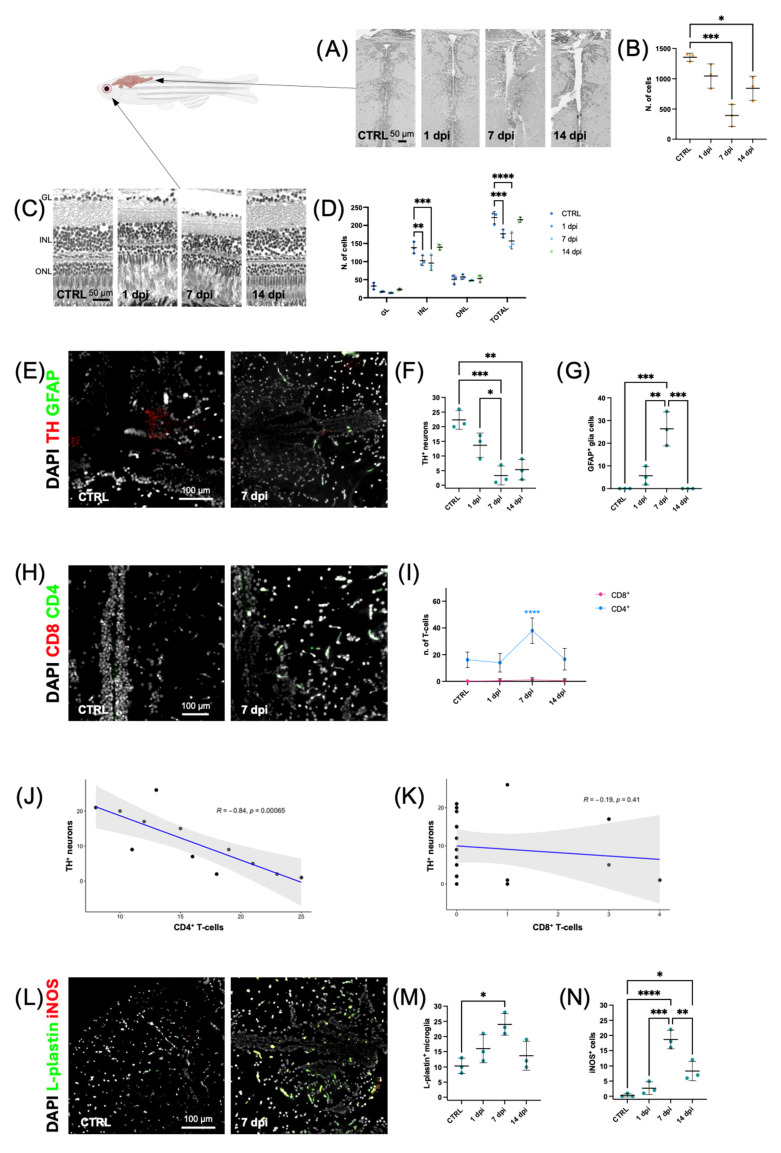
MPTP-induced neurodegeneration, astroglial activation, and T-cell infiltration in zebrafish. (**A**) Representative H&E staining of zebrafish brain sections showing the PT in healthy controls and in MPTP-treated zebrafish at 1, 7, and 14 dpi; (**B**) Quantification of PT cell numbers revealed a reduction at 7 and 14 dpi compared to controls. Analyses were performed on at least three animals per time point using one-way ANOVA followed by Tukey’s post hoc test; (**C**) Representative H&E-stained retinal sections from control and MPTP-treated zebrafish at 1, 7, and 14 dpi, highlighting progressive structural alterations and cell loss across retinal layers; (**D**) Quantification of retinal cell numbers shows a significant reduction in the INL and in the total cell count at 1 and 7 dpi. Analyses were performed using at least three animals per time point and analyzed by two-way ANOVA followed by Tukey’s post hoc test; (**E**) Representative immunofluorescence images of zebrafish brain sections stained for DAergic neuron (TH, red) and glial cells (GFAP, green) at 1, 7, and 14 dpi following MPTP treatment. Nuclei are stained with DAPI (white); (**F**) Quantification of TH^+^ dopaminergic neurons and (**G**) GFAP^+^ glial cells at 1, 7, and 14 dpi. Data were obtained from at least three animals per time point and analyzed by one-way ANOVA followed by Tukey’s post hoc test; (**H**) Representative immunofluorescence images of the PT in control and MPTP-treated zebrafish at 7 dpi, stained for CD4^+^ (green) and CD8^+^ (red) T cells. Nuclei are stained with DAPI (white); (**I**) Quantification of CD4^+^ and CD8^+^ T-cell subpopulations across time points, analyzed by two-way ANOVA followed by Tukey’s post hoc test; (**J**,**K**) Spearman correlation analysis was performed only for CD4^+^ T cells, as CD8^+^ T cells were undetectable at all time points; (**L**) Representative immunofluorescence images of PT sections showing microglia (L-plastin, green) and pro-inflammatory microglia (iNOS, red) in MPTP-treated zebrafish at 7 dpi; (**M**,**N**) The quantification analysis revealed a significant increase in microglia and pro-inflammatory microglia at 7 dpi, followed by a decrease at 14 dpi. Statistical analysis was performed using one-way ANOVA followed by Tukey’s post hoc test. *p* < 0.05 (*), *p* < 0.01 (**), *p* < 0.001 (***), *p* < 0.0001 (****).

To reproduce PD-like neurodegeneration, adult zebrafish received IP injection of MPTP (200 μg/g), a dose previously shown to selectively damage DAergic neurons in zebrafish [12]. We evaluated the effects of MPTP at 1, 7, and 14 days post-injection (dpi), focusing on two neuroanatomical regions highly sensitive to DA injury: the posterior tuberculum (PT), which is rich in DAergic neurons [19], and the retina. At 1 dpi, H&E staining of PT brain sections showed a non-significant decrease in the number of cells compared to the untreated control group (Figure 2A,B). At 7 dpi, the number of cells in the PT area significantly decreased compared to control (*** *p* < 0.001), indicating a progressive neurodegeneration during the first week after MPTP injection (Figure 2A,B). Interestingly, the number of cells in the PT slightly increased at 14 dpi compared to 7 dpi, suggesting a partial recovery (Figure 2A,B). These results show that MPTP induces a transient neuronal damage in the zebrafish brain, followed by a regenerative process that becomes evident two weeks post-treatment. In parallel with brain neurodegeneration, the zebrafish retina exhibited layer-specific neuronal loss following MPTP exposure (Figure 2C,D). H&E staining showed a marked decrease in the number of cell nuclei in the inner nuclear layer (INL) at 1 and 7 dpi compared to the control group (** *p* < 0.01 and *** *p* < 0.001, respectively) (Figure 2D). At 14 dpi, cell numbers increased compared to 7 dpi. However, the difference was not statistically significant and reached control levels, suggesting a complete recovery of the INL. The ganglion cell layer (GC) and outer nuclear layer (ONL) remained unchanged, with no significant differences across all time points, suggesting that these retinal layers are less affected by MPTP treatment (Figure 2D). Overall, the data demonstrate that retinal neurons, particularly those in the INL, are highly susceptible to MPTP-induced damage but can regenerate within two weeks of treatment.

To better characterize the involvement of astroglial response during MPTP-induced injury to DAergic neurons, we performed immunofluorescence staining using tyrosine hydroxylase (TH) to mark DAergic neurons [20] and GFAP as a marker for reactive astrocytes [21] (Figure 2E). At 7 and 14 dpi, the number of TH^+^ neurons were significantly lower than in control animals (*** *p* < 0.001 and ** *p* < 0.01, respectively) (Figure 2F). In parallel, a significant upregulation of GFAP^+^ cells at 7 dpi compared to controls was observed (*** *p* < 0.001) (Figure 2G), suggesting a reactive astrogliosis peak. Interestingly, this glial response was followed by a significant decrease at 14 dpi compared to 7 dpi, reaching the baseline levels, indicating the resolution of glial reactivity (Figure 2G). Taken together, these results show that MPTP-induced DAergic neurodegeneration is accompanied by a transient glial activation, which peaks during the degenerative phase and as regeneration begins.

To evaluate the presence of T cell infiltration during MPTP-induced neurodegeneration, we performed immunofluorescence staining using CD3, a canonical pan-T cell marker. We showed an increase in these cells at 7 dpi, followed by a decrease at 14 dpi, reaching baseline levels (Supplementary Figure S1). To further characterize the T-cell heterogeneity we analyzed CD4^+^ and CD8^+^ subsets: Immunofluorescence analysis revealed an accumulation of CD4^+^ T cells at 7 dpi (Figure 2H). CD8^+^ T cells were consistently absent across all time points (Figure 2H,I), precluding any meaningful assessment of temporal changes or correlations with neuronal loss. Quantification confirmed a significant increase in CD4^+^ T cells at 7 dpi compared with control (**** *p* < 0.0001), 1 dpi, and 14 dpi, suggesting a role for CD4^+^ T cells during the degenerative phase (Figure 2I). CD8^+^ T cells did not show any significant variation (Figure 2H,I). To investigate the relationship between different T cell subsets and neuronal damage, we performed a correlation analysis between CD4^+^ or CD8^+^ T cell counts and the number of DAergic neurons. A significant negative correlation was observed between CD4^+^ T cells and TH^+^ neurons (R = −0.84, *p* = 0.00065) (Figure 2J), while no significant correlation was found for CD8^+^ T cells (Figure 2K). These findings suggest that CD4^+^ T cells may play a role in promoting DAergic neuron loss or be involved in the subsequent regenerative process. To assess the pro-inflammatory microglial activation during MPTP-induced neurodegeneration, we performed immunofluorescence staining for L-plastin and inducible nitric oxide synthase (iNOS) (Figure 2L). L-plastin labels microglia and other macrophage-like immune cells, whereas iNOS is a hallmark enzyme of pro-inflammatory activation, typically upregulated in reactive microglia and macrophages during neuroinflammatory processes [22,23]. Quantitative analysis revealed a non-significant increase in L-plastin-positive microglia at 1 dpi compared to the control group (Figure 2M). This was followed by a significant increase at 7 dpi (* *p* < 0.1) which was higher than both the controls and the 1 dpi group (Figure 2M). The number of iNOS-positive cells decreased significantly by 14 dpi, compared with 7 dpi (Figure 2N). The number of iNOS^+^ cells decreased significantly by 14 dpi (** *p* < 0.01) compared with 7 dpi (Figure 2N).

### 2.3. Acute and Chronic Neurodegeneration in Mouse Models of PD

Adult wild-type mice were treated with MPTP using a protocol comparable to that used in zebrafish. We analyzed MPTP-induced neurodegeneration in the ventral midbrain region in mice, which corresponds to the posterior tuberculum (PT) in zebrafish, as well as the retina at 1-, 7-, and 14-days post-injection (dpi).

**Figure 3:**
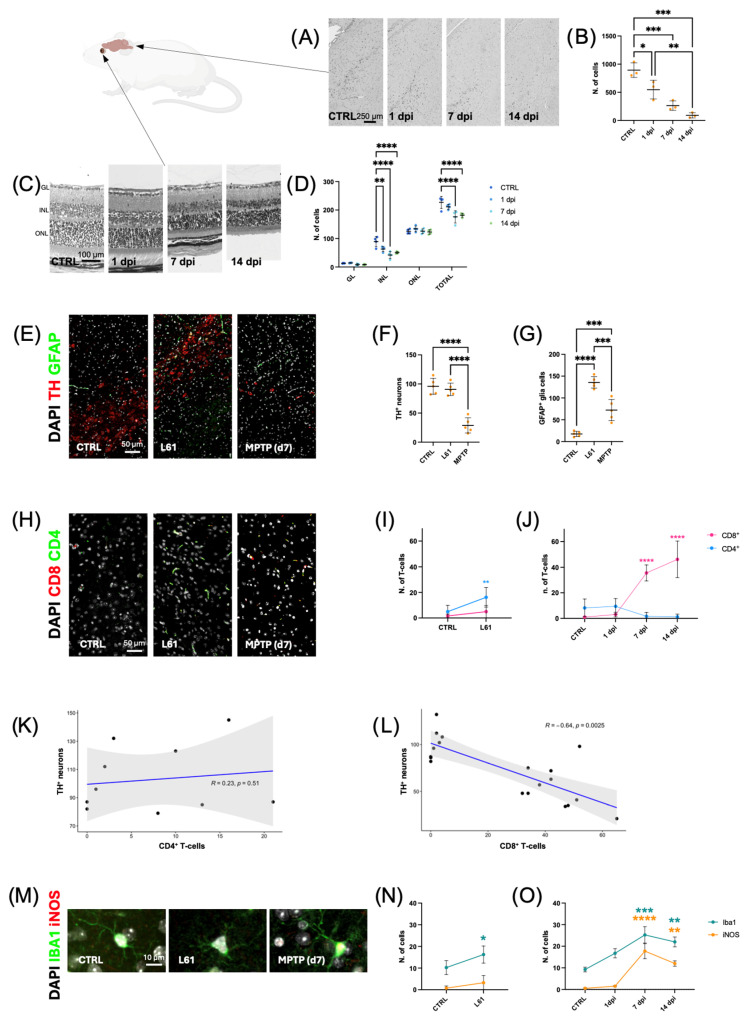
Neurodegeneration, Glial Activation, and T Cell Responses in Mouse Models of Parkinson’s Disease. (**A**) Representative H&E-stained brain sections showing the SN in control and MPTP-treated mice at 1, 7, and 14 dpi; (**B**) Quantification of cell numbers in the SN reveals a progressive reduction following MPTP administration, with the lowest counts detected at 14 dpi. Statistical significance was assessed using one-way ANOVA followed by Tukey’s post hoc test; (**C**) Representative H&E-stained retinal sections from control and MPTP-treated mice at 1, 7, and 14 dpi, showing progressive structural alterations and partial signs of recovery at 14 dpi; (**D**) Quantification of cell numbers in the GL, INL, ONL, and total retina for control and MPTP-treated mice, based on at least three animals per time point, analyzed by two-way ANOVA with Tukey’s post hoc test; (**E**) Representative immunofluorescence images of the SN from control, L61 (Thy1-aSyn) transgenic, and MPTP-treated mice. Sections were stained for dopaminergic neurons (TH^+^, red) and astrocytes (GFAP^+^, green). Nuclei were counterstained with DAPI (white). Scale bar: 50 µm; (**F**) Quantification of TH^+^ neurons and (**G**) GFAP^+^ astrocytes in L61 and MPTP-treated mice compared to controls. Data were analyzed using one-way ANOVA followed by Tukey’s post hoc test; (**H**) Representative immunofluorescence images of the SN from control, L61, and MPTP-treated mice stained for CD4^+^ (green) and CD8^+^ (red) T cells. Nuclei are stained with DAPI (white); (**I**,**J**) Quantification of CD4^+^ and CD8^+^ T-cell infiltration in L61 and MPTP-treated mice. L61 mice predominantly exhibit CD4^+^ T-cell infiltration (**I**), whereas MPTP-treated mice show a marked increase in CD8^+^ T cells (**J**). Statistical analysis was performed using two-way ANOVA followed by Tukey’s post hoc test; (**K**,**L**) Spearman correlation analyses showing the relationship between dopaminergic neuron numbers and CD4^+^ (**K**) or CD8^+^ (**L**) T-cell infiltration in MPTP-treated mice. Statistical significance is indicated in the graphs; (**M**) Representative immunofluorescence images showing microglia (IBA1, green) and pro-inflammatory enzyme (iNOS, red) in control, L61, and MPTP-treated mice; (**N**) Quantification of IBA1- and iNOS-positive cells in L61 mice shows a significant increase in the number of microglia, but no significant change in iNOS production; (**O**) Quantification of IBA1- and iNOS-positive cells in MPTP-treated mice reveals a significant increase in both microglia and pro- inflammatory microglia at 7 dpi, followed by a significant decrease in both populations at 14 dpi; (**N**,**O**) Statistical analysis was performed using two-way ANOVA followed by Tukey’s post hoc test. *p* < 0.05 (*), *p* < 0.01 (**), *p* < 0.001 (***), *p* < 0.0001 (****).

H&E staining of brain sections of MPTP-treated mice revealed a progressive loss of cellularity in the SN, initially observed at 1 dpi and further evident at 7 and 14 dpi (Figure 3A). Quantification of the number of cells showed a significant decrease at 1 dpi compared to controls (* *p* < 0.1) (Figure 3B). This reduction became more evident at 7 dpi and 14 dpi (*** *p* < 0.001) (Figure 3B). These results confirm that MPTP-induced neurodegeneration in mice progresses over time without signs of spontaneous recovery. This is in contrast to the regenerative response observed in zebrafish (Figure 3A,B), which highlights the difference in species-specific ability to restore neural tissue following MPTP-induced neurodegeneration.

Retinal morphology was examined at 1, 7, and 14 days in wild-type mice following MPTP administration. Histological analysis with H&E staining (Figure 3C,D) revealed evident tissue alterations that progressed over time. At 1 dpi, a reduction in cell density was evident, particularly affecting the INL. This loss became more pronounced by 7 dpi, with a marked thinning of the INL and disruption of the retinal structure. By 14 dpi, the retina appeared similar to that at 7 dpi, showing no clear signs of recovery or further damage. In contrast, GL and ONL cell numbers remained like the control at all time points.

To compare DAergic degeneration and astroglial reactivity elicited by two distinct PD-related insults, we analyzed the SN of L61 transgenic mice and MPTP-treated mice. Immunofluorescence analysis revealed a significant loss of TH^+^ DAergic neurons in 7 dpi MPTP-treated mice, whereas 4-month-old L61 transgenic mice showed no difference in the number of TH^+^ neurons compared to controls. Only the MPTP treatment caused a reduction in TH^+^ neuron numbers (Figure 3E,F). In parallel, we assessed astroglial activation using GFAP immunostaining (Figure 3G). Both models showed a significant increase in GFAP^+^ expression compared to the control, indicative of reactive astrogliosis. However, the astroglial response was more evident in L61 mice (Figure 3G), supporting that aSyn accumulation in this genetic model induces a stronger glial response than the acute MPTP-induced injury.

As in the zebrafish, we evaluated the presence of CD3^+^ T-cell infiltration in MPTP-treated and L61 mice by immunofluorescence and found that CD3^+^ T-cells were reduced at all time points analyzed (Supplementary Figure S2).

To further define the T-cell subtypes that infiltrate the SN during neurodegeneration, we analyzed CD4^+^ and CD8^+^ T-cells in L61 and MPTP-treated mice by immunofluorescence. Preliminary pilot experiments at 6–12 h after MPTP administration did not reveal detectable CD3^+^, CD4^+^, or CD8^+^ T-cell infiltration in either zebrafish or mice, consistent with the known delay in adaptive immune cell recruitment. Consequently, the analysis was focused on 1–14 days post-injection (dpi), capturing the onset, peak, and resolution of the acute immune response. Later time points (≥21 dpi) were not analyzed because animal welfare regulations limit prolonged survival after MPTP intoxication in mice, and in zebrafish glial and immune responses return to baseline after 14 dpi as regeneration completes. These considerations define 1–14 dpi as the biologically relevant window for cross-species comparison of acute neuroinflammatory dynamics. Results revealed a difference in T-cell subtype distribution between the two mouse models, indicating that the nature and the stage of the neurodegenerative process affect the heterogeneity of T-cell infiltration in distinct ways. In L61 mice, the majority of infiltrating T cells were CD4^+^ (** *p* < 0.01), while CD8^+^ cells were absent (Figure 3H,I). Conversely, MPTP-treated mice showed a higher number of CD8^+^ T cells, especially at 7 and 14 dpi (**** *p* < 0.0001), with only a few CD4^+^ cells observed (Figure 3H,J). To assess the potential contribution of T-cell subtypes to DAergic neurodegeneration in the MPTP model, we performed a correlation analysis between the number of infiltrating CD4^+^ and CD8^+^ T cells and the number of TH^+^ neurons in the SN of MPTP-treated mice. The analysis revealed a non-significant positive correlation between the number of CD4^+^ T cells and DAergic neurons (Figure 3K). In contrast, a significant negative correlation was observed between the number of CD8^+^ T cells and DAergic neurons (R = −0.64, *p* = 0.0025) (Figure 3L). This result indicates that higher infiltration of CD8^+^ T cells is associated with a DAergic neuron loss, supporting a potential cytotoxic role for CD8^+^ T cells in mediating neuronal damage.

To evaluate pro-inflammatory microglia, we performed immunofluorescence staining with IBA1, a pan-microglial marker, and iNOS, a marker of pro-inflammatory activation (Figure 3M). In control brain tissue, the microglia exhibited a homeostatic morphology characterized by small, compact cell bodies and thin, highly ramified processes that extended symmetrically in multiple directions. This morphology is typical of surveillant microglia in a healthy brain. No iNOS signal was detected, confirming the resting, non-activated state of these cells. In L61 mice, however, the microglia displayed morphological changes consistent with partial activation (Figure 3M). Compared to controls, the cell bodies appeared larger and adopted a more triangular or star-shaped configuration. The cellular processes were notably shorter and fewer in number, and were less branched, with some showing thickening. This intermediate phenotype resembled that of mildly activated microglia, suggesting a chronic or low-grade inflammatory state. However, iNOS expression remained minimal, indicating limited pro-inflammatory polarization. By contrast, MPTP-treated mice at 7 dpi exhibited pronounced microglial activation (Figure 3M). The cells displayed large, intensely bright cell bodies which were significantly larger than those of the control and L61 groups. The processes appeared hyper-ramified and thick, showing a preferential directional orientation rather than symmetric distribution. Notably, these cells exhibited strong iNOS immunoreactivity, confirming pro-inflammatory activation. This morphological and molecular profile is indicative of the strong neuroinflammatory response mounted by highly activated microglia following MPTP-induced neurotoxicity. Quantitative analysis revealed an increase in the number of IBA1-positive cells (* *p* < 0.1) in L61 mice compared to the control group (Figure 3N). However, iNOS-positive cells remained almost absent, indicating moderate expansion of the microglial population without significant pro-inflammatory polarization. In the MPTP model (Figure 3O), the number of IBA1-positive microglia rises progressively following MPTP injection, peaking at seven days (*** *p* < 0.001) and declining somewhat by 14 days (** *p* < 0.01). Concurrently, iNOS-positive cells were barely detectable at one dpi, exhibiting a pronounced peak at seven dpi (**** *p* < 0.0001), followed by a decline at 14 dpi (** *p* < 0.01).

### 2.4. T Cell Infiltration in the SN of PD Patients

**Figure 4:**
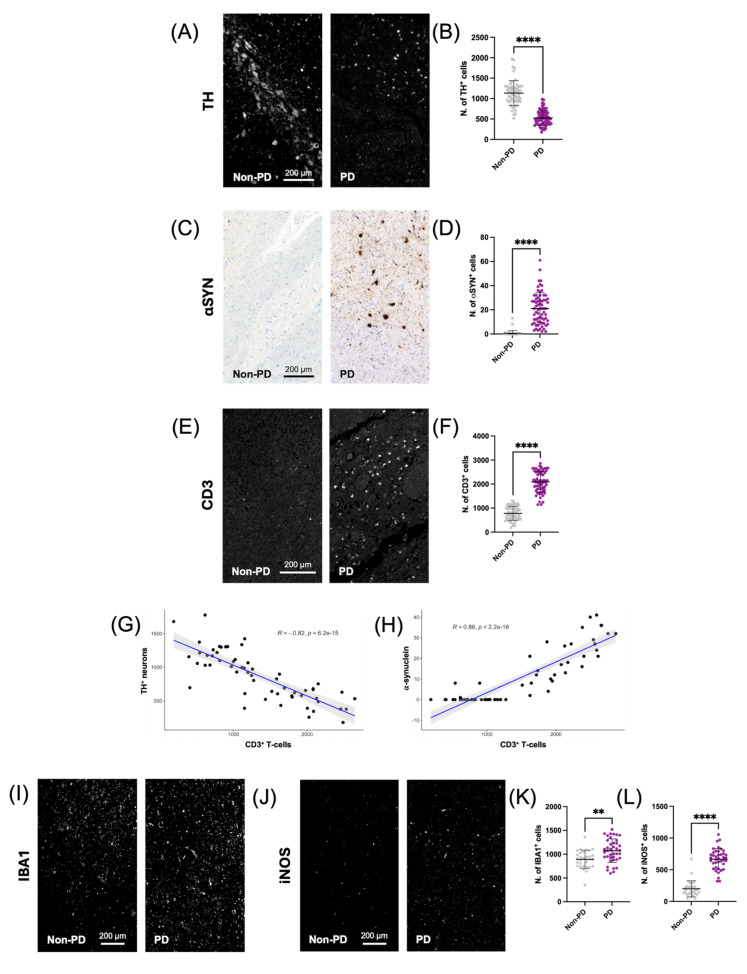
Loss of DAergic neurons, aSyn accumulation, and CD3^+^ T-cell infiltration in the substantia nigra of PD patients. (**A**) Representative immunofluorescence images of the SN from age-matched controls and PD patients stained for DAergic neurons (TH^+^, white); (**B**) Quantification of TH^+^ neurons shows a significant decrease in PD samples compared to age-matched controls (Mann–Whitney non-parametric test); (**C**) Representative immunohistochemical staining for aSyn in control and PD patient tissues; (**D**) Quantification reveals a significant increase in aSyn^+^ cells in PD patients compared to controls (Mann–Whitney non-parametric test); (**E**) Representative immunofluorescence images showing CD3^+^ T-cell infiltration in the SN of age-matched controls and PD patients; (**F**) Quantification of CD3^+^ T cells demonstrates increased infiltration in PD tissues compared to controls (Mann–Whitney non-parametric test) (**G**,**H**) Spearman correlation analyses showing the relationship between CD3^+^ T-cell infiltration and either the number of TH^+^ neurons (**G**) or aSyn accumulation (**H**) Statistical significance is indicated in the graphs; (**I**) Rappresentative immunofluorescence staining showing microglia; (**J**) Rappresentative immunofluorescence showing pro-inflammatory microglia; (**K**) Quantification of microglia (IBA1) shows an increase in positive cells in PD patients compared to age-matched controls; (**L**) Quantification of iNOS positive cells shows an increase in positive cells in PD patients compared to age-matched controls. Statistical analysis was performed using Mann–Whitney non-parametric test. *p* < 0.01 (**), *p* < 0.0001 (****).

To investigate immune involvement in PD, we examined SN sections prepared from post-mortem mesencephalon of PD patients and age-matched controls. We first assessed the extent of DAergic neurodegeneration by immunofluorescence staining on PD brains and observed a reduction and a disorganized distribution of TH^+^ neurons, compared with controls (Figure 4A). In age-matched control sections, TH^+^ neurons appeared numerous, well organized, and well distributed throughout the SN (Figure 4A). Quantitative analysis confirmed a significant decrease in TH^+^ cell numbers in PD tissues (Figure 4B) compared to the control (**** *p* < 0.0001). Next, we assessed aggregated aSyn by immunohistochemistry for alpha-synuclein at serine 129 (pSer129), a pathological modification commonly associated with Lewy body formation and enriched in aggregated species. This accumulation is implicated in neuronal dysfunction and neurodegeneration [24]. In age-matched controls, pSer129 staining was undetectable. In contrast, PD sections showed an accumulation of aSyn in the SN, with a granular pattern (Figure 4C). Quantification of aSyn^+^ cells showed a significant increase in PD samples compared to controls (**** *p* < 0.0001; Figure 4D). Together, these results confirm the loss of DAergic neurons and the pathological accumulation of aSyn in the SN of PD patients. Finally, we investigated the involvement of T-cells in PD by examining the presence of CD3^+^ T cells using immunofluorescence. We observed a marked presence of CD3^+^ cells in the SN of PD samples, while age-matched controls displayed minimal signal (Figure 4E). Quantitative analysis confirmed a significant increase in CD3^+^ T-cells in PD patients compared to controls (**** *p* < 0.0001) (Figure 4F), indicating enhanced immune cell infiltration in the diseased tissue. To determine whether T-cell infiltration was associated with DAergic neuronal loss, we performed correlation analyses. CD3^+^ T-cell density was strongly and inversely correlated with the number of TH^+^ neurons (R = −0.82, *p* = 6.2 × 10^−15^; Figure 4G), suggesting that T-cell infiltration may contribute to dopaminergic neurodegeneration. Moreover, the CD3^+^ cell count also correlated positively with aSyn levels (R = 0.86, *p* < 2.2 × 10^−16^) (Figure 4H), consistent with previous reports that aSyn aggregates could promote chemokine release, leading to T-cell recruitment [25]. Thus, our results contribute to the growing evidence that CD3^+^ T-cell infiltration is associated with both DAergic neuron loss and aSyn pathology, supporting a role for immune involvement in the pathogenesis of these core features of PD.

To assess the presence of pro-inflammatory microglia in human midbrain tissue, we performed immunofluorescence staining for IBA1 and iNOS (Figure 4I,J). As shown in the representative images, PD samples exhibit a significant increase in both IBA1-positive microglia and iNOS-expressing cells compared to age-matched non-PD controls. Quantitative analysis confirms a significant increase in IBA1^+^ (** *p* < 0.01) (Figure 4K) and in iNOS^+^ cells (**** *p* < 0.001) (Figure 4L) in PD patients, indicating an enhanced pro-inflammatory response of microglial associated with PD pathology.

## 3. Discussion

PD is characterized by the progressive loss of DAergic neurons in the ventral tier of the SN pars compacta in the midbrain, and by intraneuronal fibrillar aSyn aggregates in Lewy bodies and neurites [26]. In addition to neuronal loss and the Lewy pathology, PD shows prominent gliosis [26]. Only ~7% of the 35,502 PD–related abstracts that we systematically parsed report a multivariate link between glial reactivity (GFAP), adaptive T–cell subsets (CD4 and CD8) and DAergic neuron loss, revealing a critical gap that our cross–species analysis fills [27,28,29]. We therefore compared DAergic degeneration and T-cell infiltration across zebrafish, mouse models and human samples, with a particular focus on the brain and retina. Our findings revealed species- and model-specific differences in regenerative potential, glial reactivity, and T-cell infiltration, providing novel insights into the immunopathological mechanisms relevant to PD.

However, this study has limitations. Despite the value of complementary models such as MPTP-treated mice, MPTP-treated zebrafish, and L61 transgenic mice, none of these models alone fully captures the multifaceted complexity, heterogeneous pathology, and chronic progression characteristic of human PD. Additionally, the substantial species-specific differences in immune system organization and neurodegenerative capacity underscore the importance of careful interpretation [30]. Importantly, these models also differ fundamentally in their baseline immune architecture and in the temporal dynamics of pathology. Acute toxin-based models such as MPTP primarily induce rapid injury-associated inflammation, whereas aSyn-based transgenic models reflect chronic immune modulation that more closely resembles idiopathic PD. For example, the zebrafish’s robust ability to regenerate DAergic neurons contrasts with the largely irreversible neurodegeneration observed in mammals, potentially influencing both neuroinflammatory dynamics and tissue repair mechanisms [31]. Yet, this very difference provides a valuable counterpoint: the zebrafish offer a “ceiling condition” in which injury is naturally resolved. In zebrafish, immune–glial responses are transient and pro-resolution, marked by short-lived CD4^+^ infiltration and resolving astroglial activation. In the zebrafish model, L-plastin and iNOS show a time-dependent response, characterized by an increase of microglia and iNOS^+^ cells at 7 dpi and a decrease at 14 dpi, mirroring the trajectory of DAergic neuron loss. This result supports the presence of an acute neuroinflammatory phase followed by partial resolution, consistent with prior study of transient microglial activation after toxic injury [32]. In mammalian models, by contrast, inflammation is persistent and self-amplifying. This difference provides a framework to identify immune programs that enable neuronal recovery and for testing whether similar pathways can be reactivated in mammals and, ultimately, in human PD, a possibility that warrants further investigation. Nevertheless, as human material was limited to post-mortem substantia nigra tissue, our analysis only captures a late-stage, static view of the disease. This prevents assessment of early or evolving immune responses, which differ substantially from those observed in animal models [33]. A key limitation of the current study is the inability to perform targeted manipulation of immune pathways in mouse models. Establishing whether zebrafish-like pro-regenerative CD4^+^ responses can be activated in mammalian systems will therefore be an important focus for future investigations.

GFAP was used as the primary marker of glial activation because it represents a conserved intermediate filament upregulated in injury-responsive glial populations across vertebrates. In zebrafish, GFAP predominantly marks radial glia, which function as the main reactive and regenerative glial cells and serve as the closest functional analogue to mammalian reactive astrocytes. Although additional markers such as S100β or vimentin have been described in zebrafish, their specificity for injury-induced glial activation is limited by broader or heterogeneous expression patterns and variable modulation after neurotoxic insult. Accordingly, GFAP provides the most reliable and biologically consistent readout for comparing glial responses across the three species examined [14,34,35].

In zebrafish, MPTP exposure caused transient SN neurodegeneration and retinal damage, followed by significant recovery. Notably, TH^+^ neuron recovery in the brain and reconstitution of the INL in the retina highlight the regenerative capacity of zebrafish, consistent with a prior study demonstrating neurogenesis in this species [14]. Glial reactivity (GFAP^+^ cells) increased during the neurodegenerative phase and resolved as neuronal recovery progressed. These findings support the idea that early astrogliosis is neuroprotective, promoting neurogenesis and structural repair, as has been demonstrated previously [36], and suggest that it can facilitate rather than impede neuronal recovery. However, αSyn-driven astroglial dysfunction may promote dopaminergic loss [37]. CD3^+^ T cell infiltration mirrored this timeline, peaking at 7 dpi and declining by 14 dpi. Most of the infiltrating lymphocytes were CD4^+^ T cells, and their numbers were found to be inversely correlated with TH^+^ neurons. This suggests that an increase in CD4^+^ T cells is associated with a decrease in DAergic neurons, indicating that these cells may contribute to the early phase of neuronal degeneration. Interestingly, neuronal recovery was observed as CD4^+^ T cell numbers later declined, indicating a possible shift towards a reparative phase. Notably, CD8^+^ T cells were not detected in the zebrafish brain at any time point, indicating that cytotoxic T-cell responses are unlikely to contribute to the observed neurodegenerative or regenerative processes in this model. Although CD4^+^ T cells are not directly cytotoxic, several studies have shown that they can promote neuronal damage indirectly. This occurs through the secretion of pro-inflammatory cytokines, such as IFN-γ and TNF-α, or by activating resident glial cells (microglia and astrocytes), which can adopt a neurotoxic phenotype and further amplify inflammation [38]. However, the subsequent reduction in CD4^+^ T cells during the phase of neuronal recovery suggests that these cells may also play a beneficial regulatory role. In particular, regulatory subsets of CD4^+^ T cells are known to suppress excessive inflammation and release trophic factors that support tissue repair and regeneration [28].

While flow cytometry is a powerful approach for profiling immune-cell heterogeneity, its application to the adult zebrafish CNS is severely constrained by practical, biological, and ethical limitations. The number of T cells recoverable from each adult zebrafish brain is extremely low approximately 15 target cells per animal making cytometric quantification unreliable. This limitation is well documented in adult zebrafish neuroimmunology, where dissociation protocols result in substantial cell loss, low immune-cell yield, and poor reproducibility for rare populations [39,40]. Dissociation, staining, and washing steps further reduce viable cell numbers, substantially inflating the number of animals required to achieve statistical power across multiple timepoints. This would conflict with the principles of the 3Rs (Replacement, Reduction, Refinement) and with national regulatory guidelines governing animal experimentation. Moreover, flow cytometry would yield information overlapping with the protein-level data already obtained by immunofluorescence, without providing additional mechanistic insight. For these reasons, and in line with standard practice in adult zebrafish neuroimmunology, histological approaches remain the most feasible, biologically informative, and ethically justified method to investigate tissue-resident T-cell dynamics in the adult zebrafish brain. MPTP-treated mice, in contrast, exhibited a DAergic degeneration accompanied by a significant increase in GFAP astrocytes without neuronal recovery. The retinal structure showed only limited reorganization, consistent with the poor regenerative capacity of the adult mammalian central nervous system (CNS) [41,42], where Müller glial cells and astrocytes, principal glial cells responding to injury, mount a reactive gliosis but rarely re-enter a regenerative program [42]. Adaptive immunity also contributes. While CD3^+^ correlations with pathology markers likely reflect advanced disease states rather than causal mechanisms, their presence across divergent models supports T cell-CNS immune engagement as a translatable PD feature warranting subset-specific analysis in future human studies. Williams et al. demonstrated that depleting CD4^+^, but not CD8^+^, T cells reduced aSyn-induced neuronal loss in PD mice, suggesting that CD4^+^ T cells play a key pathogenic role in infiltrating the SN [36,38,41,42,43,44]. Our results showed that CD8^+^ T-cell numbers negatively correlated with DAergic neuron survival, consistent with reports that MPTP induces CD8^+^ T-cell infiltration and activation [45]. MPTP-treated mice exhibit a significant increase in IBA1^+^ microglia and iNOS induction at 7 dpi, followed by a decrease in both markers at 14 dpi, which is indicative of an acute inflammatory response. Consistent with our previous data using the MPTP model, these results suggest that MPTP triggers pro-inflammatory activation of microglia coinciding with the decline of DAergic neurons. This inflammatory ‘burst’, characterized by increased cell numbers, morphological changes, and the upregulation of effector enzymes such as iNOS, has been described as a key contributor to the amplification of neuronal damage in the SN. In contrast, the subsequent partial normalization of microglia reflects the onset of innate resolution mechanisms [46]. Future TH immunostaining studies could elucidate dopaminergic amacrine-specific responses to MPTP and their relative contribution to layer-wide retinal pathology and regeneration.

The L61 transgenic model exhibits a slower, aSyn-driven loss of DAergic neurons, accompanied by astrogliosis, similar to that seen in PD patients [15]. In the L61 mouse model, the morphology of the microglia is activated, with an increased number of IBA1^+^ cells and minimal iNOS expression. This is indicative of low-grade chronic activation rather than a fully polarised pro-inflammatory state. This inflammatory profile is consistent with the slow and progressive nature of aSyn-driven pathology, in which sustained but non-cytotoxic microglial activation is observed. While detailed characterization of the microglia in Thy1-aSyn L61 mice is limited, previous studies have described the gradual development of α-synucleinopathy accompanied by age-dependent neuroinflammation, which supports this interpretation. Consistent with observations from other aSyn-based models, in vitro experiments have shown that prolonged exposure to aSyn aggregates induces persistent yet heterogeneous activation states. Our data further indicate that L61 microglia maintain a mild inflammatory signature reflective of the underlying progressive pathology [17,47]. Our results demonstrated that T-cell infiltration was predominantly composed of CD4^+^ T-cells, with no detectable CD8^+^ population. Importantly, the presence of CD4^+^ T cells at early stages (e.g., 4 months), prior to the onset of DAergic neuron loss, which typically occurs after 14 months, suggests a modulatory rather than cytotoxic role. This pre-degenerative inflammatory state is supported by literature showing CD4^+^ T-cell accumulation beginning around 5–6 months in the L61 model [17]. Similar neuroprotective functions of CD4^+^ T cells have been described in other chronic neurodegenerative conditions, such as amyotrophic lateral sclerosis [48]. While brain-focused DA/glial/T-cell profiling provides direct PD neuropathology correlates, our complementary retinal H&E analyses reveal tissue-level vulnerability patterns. Subtype-specific retinal immunofluorescence (TH^+^, Müller glia, resident microglia) warrants dedicated future investigation.

Our data delineate a trajectory from transient regeneration-permissive CD4^+^ responses in zebrafish to persistent GFAP^+^ astrogliosis and CD8^+^ cytotoxic recruitment in the mouse model. Although the present study did not include dedicated cell-proliferation or lineage-tracing assays, the zebrafish data provide indirect but clear evidence of regeneration. After MPTP exposure, both TH^+^ neuronal counts and retinal cellularity showed partial recovery by 14 dpi, paralleled by the normalization of GFAP^+^ glial reactivity and CD4^+^ T-cell infiltration. These dynamics are consistent with the established regenerative time course described in prior zebrafish studies [13,14,31]. Given that our main objective was to contrast the immune–glial landscape across species, detailed molecular characterization of the regenerative process was beyond the present scope but will be addressed in future work.

Human SN analyses confirmed the cardinal PD features of DAergic neurodegeneration and aSyn aggregation [24,26]. CD3^+^ T-cell infiltration correlated positively with aSyn pathology and inversely with TH^+^ neurons counts, linking T-cell presence to both major PD hallmarks. Although we did not distinguish T-cell subsets, these findings are consistent with reports that misfolded aSyn can activate microglia and drive chemokine release, fostering recruitment of T-cells, including cytotoxic CD8^+^ populations that have been shown to damage DAergic neurons in PD [29,47,49,50,51,52,53,54,55]. Moreover, the concurrent increase in IBA1^+^ microglia and iNOS^+^ cells in the midbrain of PD patients compared with controls supports the hypothesis that persistent microglial neuroinflammation contributes to disease progression. This interpretation is consistent with a previous study showing activated microglia in the SN of PD patients, together with sustained production of pro-inflammatory cytokines and reactive nitrogen species that are considered key drivers of DAergic neuron vulnerability and ongoing neurodegeneration [7].

The divergent T-cell signatures observed across models are likely shaped by multiple factors, including differences in the onset and progression of injury, variations in antigenic load, and the distinct inflammatory milieus characteristic of each system. Importantly, our comparisons were aligned to analogous phases of model-relevant immune activity rather than identical chronological time points, acknowledging the intrinsic biological distinctions between acute, chronic, and regenerative paradigms. Given these differences in injury kinetics, variations in T-cell composition may therefore reflect the specific phase of injury rather than a species-dependent regenerative capacity. Our data are inherently correlative and do not establish direct causality. Without peripheral immune profiling (e.g., blood, spleen, or lymphoid tissues), we also cannot determine whether CNS infiltration reflects selective central recruitment or broader systemic inflammatory priming. Future mechanistic studies, including temporally matched longitudinal analyses within each model, peripheral immune characterization and pathway manipulation in mammalian systems, will be required to distinguish temporal effects from species-specific ones, and to determine whether the pro-resolution immune programs observed in zebrafish can be reactivated in mammals. Integrating peripheral T-cell phenotyping, antigen-specific activation assays and chemokine/cytokine profiling in serum and spleen is essential for distinguishing systemic inflammatory priming from CNS-specific mechanisms of T-cell recruitment. We interpret the cross-species alignment of immune–glial states and DAergic outcomes as being associative rather than causal. Accordingly, our conclusions are hypothesis-generating and future interventional studies are necessary to establish the mechanism.

In our MPTP models, acute neurotoxin-induced DAergic necrosis and associated blood–brain barrier (BBB) disruption, via mechanisms such as MMP-3 upregulation and cytokine-mediated tight junction breakdown, likely serve as the primary attractants for peripheral immune cell infiltration, preceding overt glial activation [56,57]. This sequence is supported by the temporal profile observed: neuronal loss and microglial priming peak prior to maximal T-cell accumulation at 7 dpi (Figure 2 and Figure 3). The divergent GFAP reactivity kinetics between MPTP zebrafish (transient peak at 7 dpi, resolving by 14 dpi) and mice (sustained elevation) further suggest a positive feedback loop in mammals, where initial glial responses amplify chemokine production (e.g., via TNF-α, IL-1β), sustaining CD8^+^ T-cell recruitment and chronic neuroinflammation that exacerbates pathology [58,59]. This model reconciles our focus on CNS-resident dynamics with the need for peripheral context, warranting future studies integrating BBB markers (e.g., claudin-5) and systemic profiling.

While our findings highlight potential immune–glial pathways relevant to PD pathogenesis, we acknowledge that the present study does not directly test immunotherapeutic interventions in human systems. However, our data are correlative and do not establish direct causality, recent clinical studies have shown that enhancing regulatory T-cell responses with recombinant human GM-CSF (sargramostim; NCT03790670) can modulate peripheral immune activity and may exert neuroprotective effects in PD [60,61]. This clinical evidence supports the biological plausibility of T-cell involvement in neuronal maintenance and degeneration, as observed across our experimental models.

In summary, our cross-species analysis reveals that transient, pro-regenerative astroglial activation observed in zebrafish is in contrasts with the persistent inflammation and dopaminergic loss seen in mammalian models and human PD [29,44,49].

## 4. Materials and Methods

### 4.1. Bibliographic Data Mining

We queried PubMed programmatically using the NCBI Entrez E-utilities for four experimental contexts: zebrafish, mouse, human, and cross-species comparative models of PD.

Each query was executed within an adaptive year-window recursion that partitions the global time span from 1 January 1960 to 31 December 2025. All PMIDs were harvested in batches to guarantee completeness and server compliance, yielding 35,502 unique PMIDs. For every PMID, we retrieved full MEDLINE XML and normalised title, abstract, MeSH, keyword, journal, authors and publication year into a relational dataframe (pandas v2.2) (Supplementary Table S1).

The structured neuro-immunological information was extracted from the complete abstract set using DeepSeek–r1 (7-B, instruction-tuned), executed locally via Ollama (Ollama, Inc., San Francisco, CA, USA) on a CUDA 12.4 GPU (Quadro T2000, NVIDIA Corporation, Santa Clara, CA, USA). A deterministic, few-shot prompt constrained the model to populate a nine-field template (context, species, GFAP-CD4/CD8 relations, Dopaminergic neuron loss, multivariate pattern, GFAP reliability as a astroglial marker). Responses were parsed with regex against pre-validated patterns; if ≥5 fields were “not mentioned” the abstract was automatically re-submitted with a stricter fallback prompt. The pipeline yielded 380 high-confidence structured records. Outputs underwent (i) automatic sanity checks (duplicate abstracts, illegal field values, missing keys) and (ii) manual review of a 10% stratified random sample per species. Discrepancies were corrected or flagged, concordance with human curators. By combining rule-based post-processing, duplicate removal and critical human validation, we minimised recency biases intrinsic to LLMs, ensuring a robust evidence base for downstream statistical and clustering analyses. The code is provided in the Supplementary Materials.

### 4.2. Zebrafish Studies

Adult zebrafish of both sexes (AB strain) were kept at the animal facility of the Department of Anatomy, University of Bern. Only adult zebrafish (~1 year old) were used in this study. They were maintained under standard conditions in tank water at 26.5 °C and raised on a 14/10 h light/dark cycle, as previously described [62,63]. Fish were fed dry food twice daily (GEMMA Micro 300; Westbrook, ME, USA) and TetraMin Tropical Flakes (Delphin-Amazonia AG, Münchenstein, Switzerland) once per day, as well as Artemia salina once per day. During experiments, animals were kept in tank water.

Adult zebrafish received intraperitoneal (IP) MPTP to induce degeneration of DA neurons (Sigma-Aldrich, St. Louis, MO, USA). MPTP was freshly dissolved in sterile saline to a final concentration of 10 mg/mL. The injection volume was adjusted according to body weight, with each fish receiving 200 µg MPTP per gram body weight in a total volume of 10 µL per fish [12,63,64].

Prior to injection, fish were anesthetized with 0.16 mg/mL ethyl 3-aminobenzoate methanesulfonate salt (Tricaine; Sigma-Aldrich, Buchs, Switzerland) dissolved in tank water until loss of the righting reflex. Anesthetized fish were placed ventral side up on a moist sponge for stabilization. Using a 30-gauge insulin syringe, the MPTP solution was injected into the intraperitoneal cavity at the ventral midline, just posterior to the pelvic girdle, at an angle of approximately 45° to the body axis [11,20]. Control animals received an equivalent volume of sterile saline.

Following injection, fish were transferred to a recovery tank containing fresh system water and monitored until full recovery from anesthesia.

### 4.3. Mouse Studies

Adult C57BL/6J mice (Charles River Germany, Sulzfeld, Germany), both male and female, aged 6–9 weeks, were used in all experiments. Animals were housed under standard conditions in individually ventilated cages (IVCs) with a 12-h light/dark cycle in a temperature-controlled animal facility at the Central Animal Facilities of the University of Bern. Mice had ad libitum access to standard laboratory chow and water.

To induce DA neurodegeneration, mice received IP injections of MPTP at a dose of 40 mg/kg body weight, dissolved in sterile saline and administered in a total volume of 10 mL/kg [65]. Control animals received an equivalent volume of sterile saline. All experimental procedures were approved by the governmental authorities of the Canton of Bern (BE 28/2024).

Paraffin-embedded 5 μm sections from 4-month-old Thy1-aSyn (L61) transgenic mice, which overexpress human wild-type aSyn under the Thy1 promoter, were kindly provided by Prof. Thomas Montine (Stanford University, Stanford, CA, USA) for comparative histological analysis.

### 4.4. Human Studies

Tissue microarrays (TMAs) of selected midbrain areas were produced as previously described [66,67,68]. Briefly, archived human midbrain paraffin-embedded tissue was stained with hematoxylin–eosin (H&E) to identify regions of the left and right substantia nigra pars compacta (SNc) suitable for TMA construction. Tissue cores (2 mm diameter) were punched from these regions and transferred to recipient blocks, each containing approximately 40 samples. The first three punches in each TMA consisted of non-neuronal tissues (kidney or liver) as controls. Age-matched and PD samples were placed in separate blocks and sorted by collection date. TMA blocks were sectioned at 2.5 µm. The first section was stained with H&E, and the second was stained with aSyn to verify correct annotation and exclude aSyn pathology in age-matched samples.

### 4.5. Histology and Cell Quantification

Morphological analyses were performed on H&E-stained sections of eyes and brains collected at 1-, 7-, and 14-day post-injection (dpi) from C57BL/6J mice and zebrafish.

Following fixation in 4% paraformaldehyde at 4 °C overnight, tissues were dehydrated in a graded ethanol series, cleared in xylene, and embedded in paraffin. Serial frontal sections were cut at a thickness of 5 µm using a rotary microtome, mounted on glass slides, deparaffinized, and rehydrated through a series of descending alcohol concentrations to water. H&E staining (hematoxylin from Sigma, St. Louis, MO, USA, and eosin from Roth, Karlsruhe, Germany) was performed according to established protocols [69].

Quantification of cell nuclei was carried out by manual counting on H&E-stained sections using a light microscope. In both zebrafish and mouse eyes, a region of interest (ROI) measuring 200 µm in width was defined in the central retina, and all clearly identifiable nuclei within this region were counted [69]. For mouse brain sections, the SN was identified based on anatomical landmarks, and all nuclei within this area were counted [70]. In zebrafish brain sections, the PT, considered functionally analogous to the mammalian SN [71], was similarly identified and analyzed. For each model, three non-overlapping sections were selected for analysis, and the average number of nuclei per region was calculated.

### 4.6. Immunofluorescence

Paraffin sections (5 μm) were utilized for immunofluorescence. Antigen retrieval was achieved by incubating the sections in Tris–EDTA (pH 9.0) or Citrate buffer (pH 6.0) with 0.05% Tween-20 for 4 min, followed by cooling at room temperature for a minimum of 30 min. Blocking was performed for 1 h in Tris-buffered saline (TBS; pH 7.6) containing 5% goat normal serum (DAKO, Agilent Technologies, Baar, Switzerland) and 1% bovine serum albumin (Sigma-Aldrich). The sections were incubated overnight at 4 °C with primary antibodies diluted in TBS. Primary antibodies used in this study were rabbit anti-tyrosine hydroxylase (TH, MAB5280; 1:1000; Millipore, Billerica, MA, USA) rabbit anti-aSyn (phospho S129; ab51253; 10 μg/mL; Abcam, Cambridge, UK), rabbit anti-glial fibrillary acidic protein (GFAP; OPA1-06100; 1:200; Thermo Scientific, Wilmington, DE, USA), mouse anti-CD3 antibody (ab237721; 1:2000; Abcam), mouse anti-CD4 antibody (MA5-15775; Invitrogen, Carlsbad, CA, USA) and rabbit anti-CD8 antibody (ab217344, 1:1000; Abcam), rabbit anti–α leucocyte-specific actin-bundling protein L-plastin (1:500; kindly provided by Jaźwińska, University of Fribourg, Fribourg, Switzerland, as previously described [72]), rabbit polyclonal anti-IBA1 (019-19741; 1:500; Wako, Osaka, Japan), and mouse monoclonal anti-inducible nitric oxide synthase (iNOS; MAB9502, 1:200; R&D Systems, Minneapolis, MN, USA).

Sections were then washed for 20 min and stained at room temperature with the respective secondary Antibodies (1:500; Alexa Fluor 488 or 594, Abcam, Cambridge, UK) diluted in TBS with 1% BSA for 1 h at room temperature. Cell nuclei were counterstained using Vectashield mounting media with 4′,6-diamidino-2-phenylindole (DAPI; Vector Labs, Burlingame, CA, USA).

For each staining series, sections were processed in parallel with omission of the primary antibody to exclude non-specific binding of secondary antibodies and autofluorescence. No detectable signal was observed in these negative control sections.

Immunostaining analyses were independently performed by two blinded investigators, with data included only when inter-observer concordance exceeded 90%; discrepancies were resolved by consensus. Quantification was ROI-based rather than stereological. Although anatomical ROIs and blinded dual scoring were used to reduce variability, unbiased stereology would be required for absolute neuronal estimates. Accordingly, our results rely on within-model relative differences rather than absolute cell numbers.

### 4.7. Statistics

All quantifications were performed by two independent, blinded observers using ImageJ/Fiji software version 2.9.0 (National Institutes of Health, Bethesda, MD, USA) [73] to minimize bias. Data were assessed for normality prior to statistical analysis. When normality was confirmed, parametric tests were applied, including unpaired t-tests or one-way ANOVA with Tukey’s post hoc test for multiple comparisons. If normality was not met, non-parametric tests such as the Mann–Whitney U test were used, and results are reported as median values. For longitudinal data, linear mixed-effects models were employed. The specific statistical tests used are detailed in the corresponding figure legends and text. Statistical significance was defined as *p*  ≤  0.05. All analyses were conducted using GraphPad Prism 9.4.1 (GraphPad Software, Boston, MA, USA). Correlation analyses were performed using Spearman’s correlation matrices generated with the corrplot function from the gplots package (version 3.0.1), and pairwise correlations between two variables were visualized using the ggscatter function from the ggpubr package (version 0.6.0), in R version 3.5.1.

## 5. Conclusions

Our data reveal model-specific differences in neuronal loss, astroglial reactivity, and T-cell infiltration. Further studies using higher-resolution approaches will be necessary to fully characterize T-cell heterogeneity and its dynamic contribution to neurodegeneration and recovery beyond the major subsets examined here across species. While zebrafish exhibited a DAergic neurodegeneration followed by regeneration, mouse models and human tissues showed DAergic neurodegeneration with no evidence of recovery.

Across 35,502 PD abstracts, <10% connect GFAP^+^ astrogliosis, CD4^+^ or CD8^+^ T–cell dynamics and DAergic loss [27,28]. Our results help address this gap, showing that zebrafish mount a brief, CD4–dominated response after MPTP-induced degeneration, concomitant with a transient reactive gliosis [14,36]. In contrast, mammalian models and human SN display persistent GFAP^+^ reactivity and persistent T-cell presence [43,45,49]. These findings point to the dual nature of immune responses in neurodegeneration, with distinct temporal trajectories and functional outcomes depending on the model. However, our data are correlative and centered on tissue-resident immunity; without peripheral immune profiling, we cannot determine whether CNS infiltration reflects selective central recruitment or broader systemic priming. Future mechanistic studies, including temporally matched longitudinal sampling, peripheral T-cell phenotyping [29,44] and cytokine profiling, and targeted pathway manipulation in mammalian systems, will be required to disentangle temporal from species-specific effects and to test whether pro-resolution immune programs observed in zebrafish can be reactivated in mammals.

This work provides a cross-species comparative framework that integrates models with distinct pathogenic mechanisms and regenerative capacities. By directly contrasting zebrafish, mouse, and human PD samples, our study highlights both conserved and divergent patterns of T-cell infiltration and astroglial activation. This integrative approach expands current immune-neurobiological knowledge by revealing how immune–glial interactions is shaped by species-specific regenerative potential, thereby offering a translational context for future mechanistic studies.

## Data Availability

The original contributions presented in this study are included in the article/Supplementary Materials. Further inquiries can be directed to the corresponding author.

## Supplementary Materials

The supporting information can be downloaded at: https://www.mdpi.com/article/10.3390/ijms27020965/s1.

## Abbreviations

The following abbreviations are used in this manuscript:

PD	Parkinson’s disease
DAergic	dopaminergic
aSyn	α-synuclein
SN	substantia nigra
MPTP	1-methyl-4-phenyl-1,2,3,6-tetrahydropyridine
GC	ganglion cell layer
ONL	outer nuclear layer
INL	inner nuclear layer
PT	posterior tuberculum
TH	tyrosine hydroxylase
GFAP	glial fibrillary acidic protein
dpi	days post-injection
L61	hy1-aSyn Line 61 transgenic mouse model
H&E	hematoxylin and eosin staining
TMA	tissue microarrays
BSA	bovine serum albumin
TBS	tris-buffered saline
PMIDs	PubMed Identifiers
pSer129	phospho-serine 129 (of alpha-synuclein)
DAPI	4′,6-diamidino-2-phenylindole
CNS	central nervous system
