# Supplementary material for: Comparative Analysis of T-Cell Signatures and Astroglial Reactivity in Parkinson’s Pathology Across Animal Models with Distinct Regenerative Capacities
Source: Int J Mol Sci. 2026 Jan 18;27(2):965. doi: 10.3390/ijms27020965 (PMC12841556; doi:10.3390/ijms27020965)
Supplement: Supplementary file 1 [file ijms-27-00965-s001.zip › Supplementary files.pdf]

SUPPLEMENTARY MATERIAL

T-Cell involvement in DA neurodegeneration in Zebrafish

To assess T-cell infiltration following MPTP-induced injury, immunofluorescence for CD3^{+} T cells was performed in the PT of healthy control and MPTP-treated zebrafish. Results revealed a significant increase in the number of CD3^{+} T cells at 7 dpi compared to both control and 1 dpi (**Supplementary Figure S1A,B),** suggesting that CD3^{+} T cell infiltration is associated with DA neuron loss. Interestingly, at 14 dpi, CD3^{+} T-cell numbers significantly decreased compared to 7 dpi, reaching the baseline, suggesting that T cell recruitment is linked to the acute phase of neurodegeneration and may resolve as the tissue begins to recover. A significant negative correlation **(Supplementary Figure S1C)** was observed between the number of CD3^{+} T cells and DA neurons ($R = –0.84$, $p < 0.0001$). This negative relationship suggests that higher levels of CD3^{+}T-cell infiltration are associated with a reduction in DA neurons, supporting the hypothesis that T cells might play an active role in promoting neurodegeneration and neuronal loss in zebrafish.

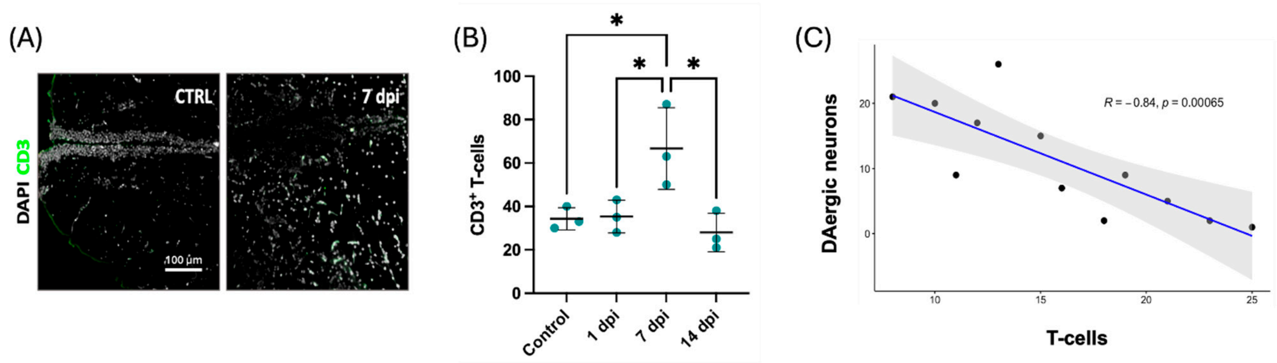

Supplementary Figure S1: CD3^{+} T cells infiltration during MPTP-induced neurodegeneration. (A) Representative immunofluorescence images of the posterior tuberculum showing CD3^{+} T cells (green) in control and MPTP-treated zebrafish at 7 dpi. Nuclei are stained with DAPI (white). **(B)** Quantification of CD3^{+} T cells at different time points after treatment. **(C)** Correlation between DA neurons and the number of T-cells recruited in the MPTP-treated zebrafish brain. Statistical differences are shown in the graphs.

T cells immune response in L61 and MPTP treated mice

To investigate T-cell infiltration in different models of PD, we quantified CD3^{+} T cells in the PT of L61 transgenic mice and MPTP-treated mice. In L61 mice, we observed a significant increase in CD3^{+} T cells compared to wild-type controls **(Supplementary Figure S2A)**, indicating T-cell recruitment in response to chronic α-synuclein accumulation. Similarly, in the MPTP model, CD3^{+} T-cell infiltration into the PT was observed to follow a time-dependent pattern. At 1 dpi, a slight but not statistically significant increase in CD3^{+} cells was detected compared to controls. This infiltration increased at 7 dpi and reached the maximum level at 14 dpi **(Supplementary Figure S2B**), indicating a gradual accumulation of T cells over time. These results suggest that the adaptive immune response

becomes stronger as neurodegeneration advances, with the highest level of T-cell infiltration occurring during the later stages of the neuronal damage. To explore whether T-cell presence was associated with DA neuron loss, we performed correlation analyses between CD3$^+$ T-cells and the number of TH$^+$ neurons. In L61 mice **(Supplementary Figure S2C)**, no correlation was observed between T-cell presence and DA neuronal loss. Conversely, in the MPTP model **Supplementary Figure S2C)**, we observed a significant negative correlation ($R = -0.64$, $p = 0.0025$), indicating that higher numbers of CD3$^+$ T cells were associated with a stronger reduction in TH$^+$ neurons **(Supplementary Figure S2D)**. These findings support the hypothesis that CD3$^+$ T cells may contribute to neurodegeneration in the MPTP model, while their role in the L61 model appears to be more complex and not linked to neuronal loss.

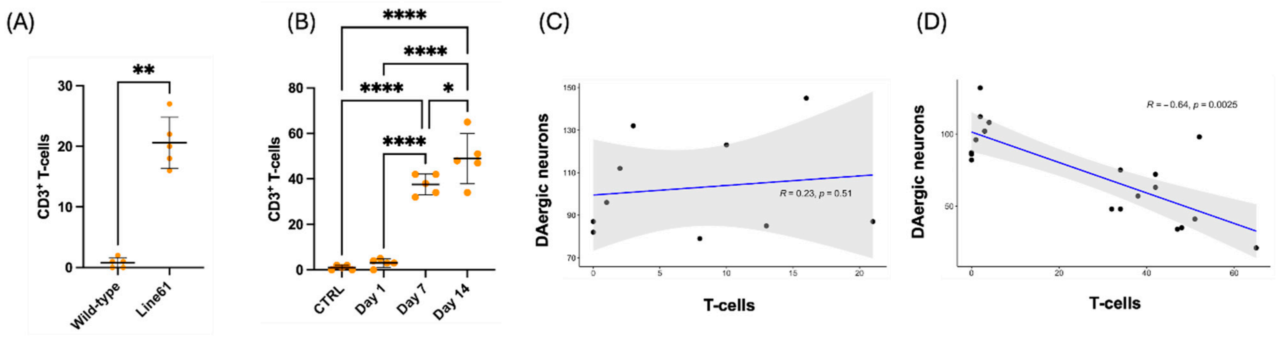

Supplementary Figure S2: CD3$^+$ T-cell infiltration and correlation with DA neuron loss in L61 and MPTP models. (A) Quantification of CD3$^+$ T cells in the PT of L61 and **(B)** MPTP-treated mice. **(C)** Correlation between CD3$^+$ T cells and TH$^+$ neurons in L61 and **(D)** MPTP-treated mice. Statistical differences are shown in the graphs.

Bibliographic data mining

Supplementary Table S1. PubMed interrogation strategy. Extracted metadata (PMID, Title, Abstract, Journal, Year, Authors, MeSH terms, Author Keywords) underwent rigorous deduplication (PMID & text hash) and harmonization (NA replaced by pd.NA) (file 01_PMID_PCA_TD-IDF.ipynb and the output df_corpus.pkl provided in the supplementary material) to produce a consistent corpus (df_corpus.pkl).

Model	PubMed search strategy*
Zebrafish	(Parkinson Disease[MeSH] OR parkinson*[tiab]) AND (zebrafish[MeSH] OR zebrafish[tiab] OR Danio rerio[tiab]) AND (MPTP[tiab] OR "dopaminergic neuron"[tiab] OR retina[tiab] OR regeneration[tiab] OR "T cell"[tiab] OR astrocyte[tiab] OR microglia[tiab] OR immun*[tiab]) AND ("1960/01/01"[PDAT] : "2025/12/31"[PDAT])

Mouse	(Parkinson Disease[MeSH] OR parkinson*[tiab]) AND (mouse[MeSH] OR mice[tiab] OR murine[tiab]) AND (MPTP[tiab] OR "alpha-synuclein"[tiab] OR L61[tiab] OR Thy1[tiab] OR transgenic[tiab]) AND ("T cell"[tiab] OR lymphocyte[tiab] OR immun*[tiab] OR microglia[tiab] OR astrocyte[tiab] OR retina[tiab]) AND ("1960/01/01"[PDAT] : "2025/12/31"[PDAT])
Human	(Parkinson Disease[MeSH] OR parkinson*[tiab]) AND (human[MeSH] OR human[tiab] OR patient[tiab]) AND ("post-mortem"[tiab] OR "substantia nigra"[tiab] OR immunohistochemistry[tiab] OR "T cell"[tiab] OR lymphocyte[tiab] OR immun*[tiab] OR microglia[tiab] OR astrocyte[tiab] OR "alpha-synuclein"[tiab] OR Lewy[tiab]) AND ("1960/01/01"[PDAT] : "2025/12/31"[PDAT])
Comparative	(Parkinson Disease[MeSH] OR parkinson*[tiab]) AND (zebrafish[tiab] OR Danio rerio[tiab] OR mouse[tiab] OR mice[tiab] OR murine[tiab] OR human[tiab]) AND (model*[tiab] OR comparative[tiab]) AND ("T cell"[tiab] OR lymphocyte[tiab] OR immun*[tiab] OR microglia[tiab] OR astrocyte[tiab] OR "alpha-synuclein"[tiab]) AND ("1960/01/01"[PDAT] : "2025/12/31"[PDAT])
